# Supplementary material for: Dynamic Plasma Lipidomic Analysis Revealed Cholesterol Ester and Amides Associated with Sepsis Development in Critically Ill Patients after Cardiovascular Surgery with Cardiopulmonary Bypass
Source: J Pers Med. 2022 Nov 3;12(11):1838. doi: 10.3390/jpm12111838 (PMC9693300; doi:10.3390/jpm12111838)
Supplement: Supplementary file 1 [file jpm-12-01838-s001.zip › supplementary files/supplementary figures.pdf]

Supplementary Figure S1

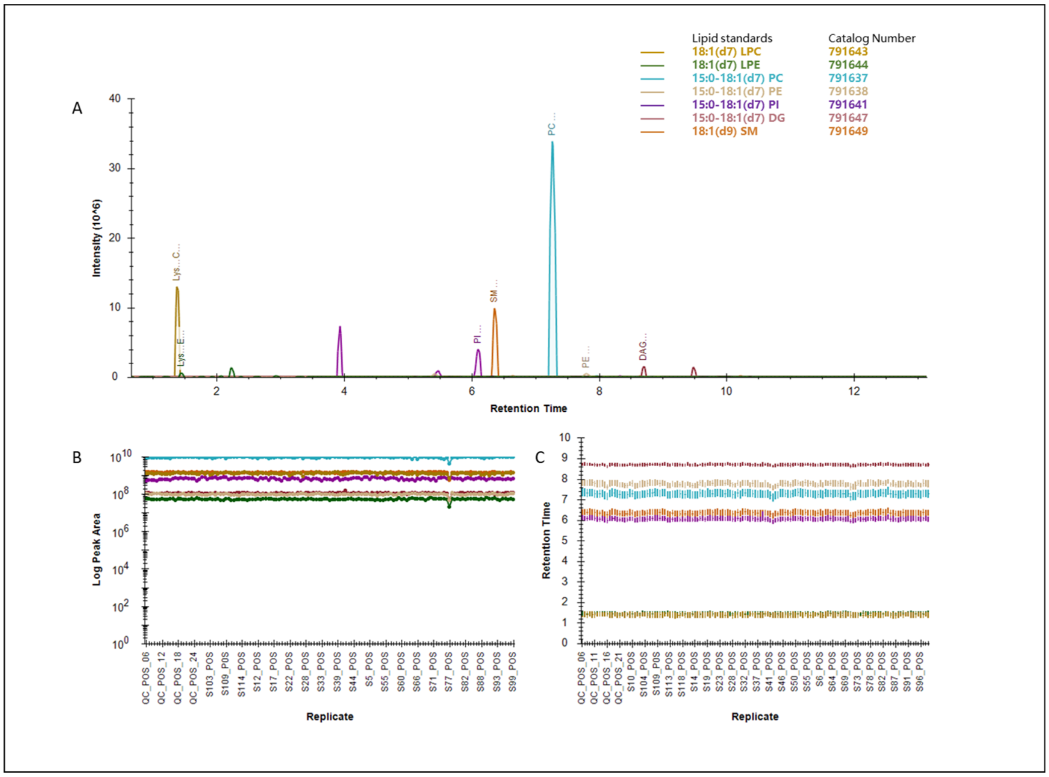

Figure S1. Seven isotope labeled internal standard of lipids for monitoring the stability of experiment operation. Exactracted ion chromatogram of seven IS(A). Peak area of seven IS in 139 samples(B). Retention time of seven IS in 139 samples (C).

Supplementary Figure S2

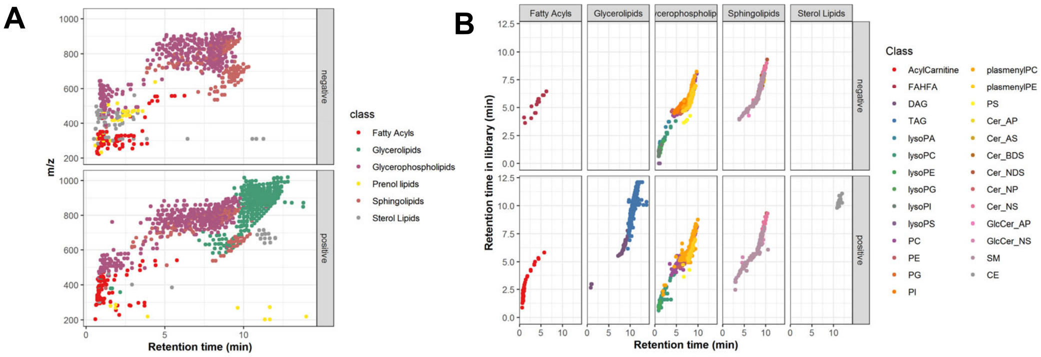

Figure S2. A global view of lipidome identification. Lipids distribution in retention time level

and m/z level(A). A comparison of retention time of lipids in our experiment to those in Lipidblast library (B).

Supplementary Figure S3

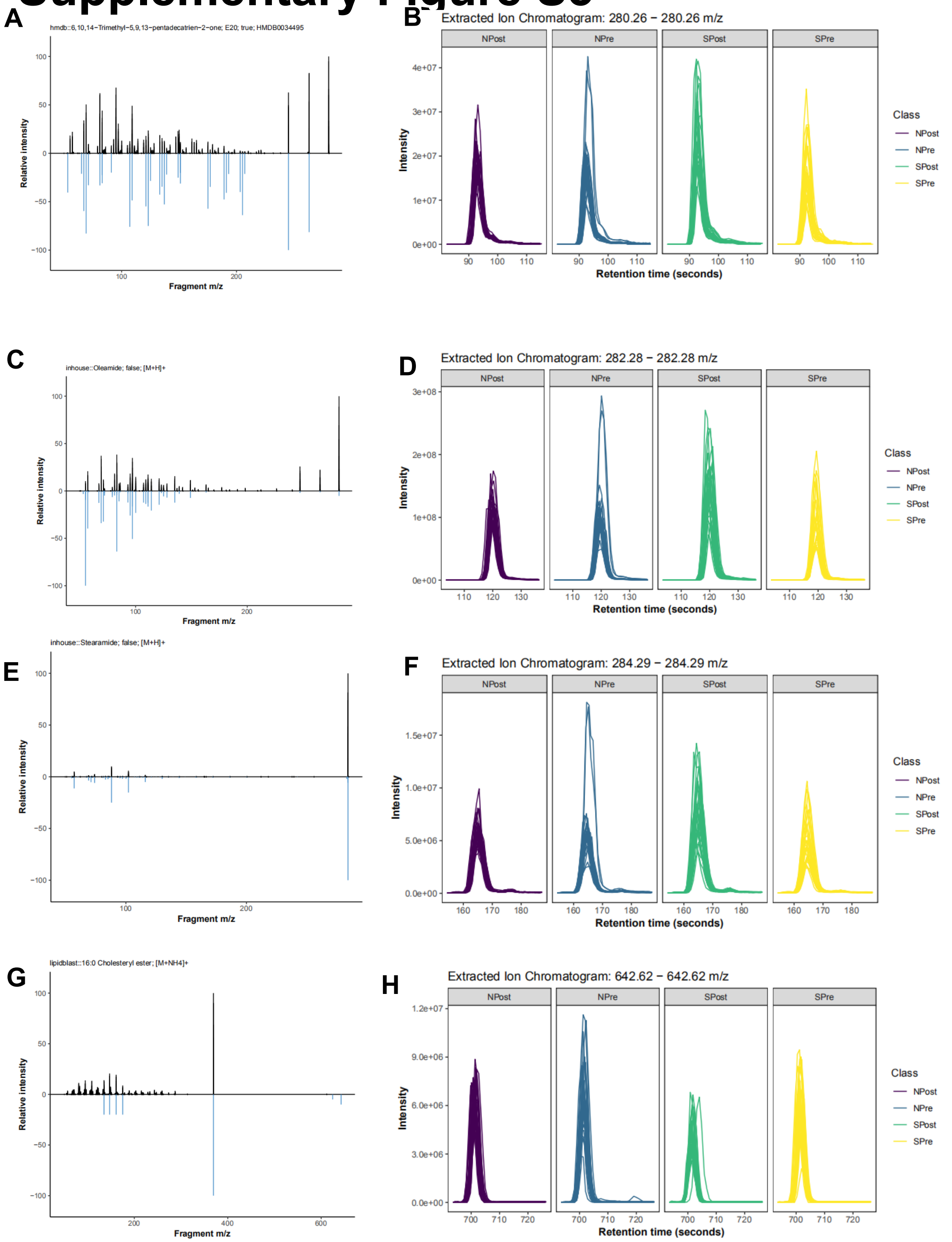

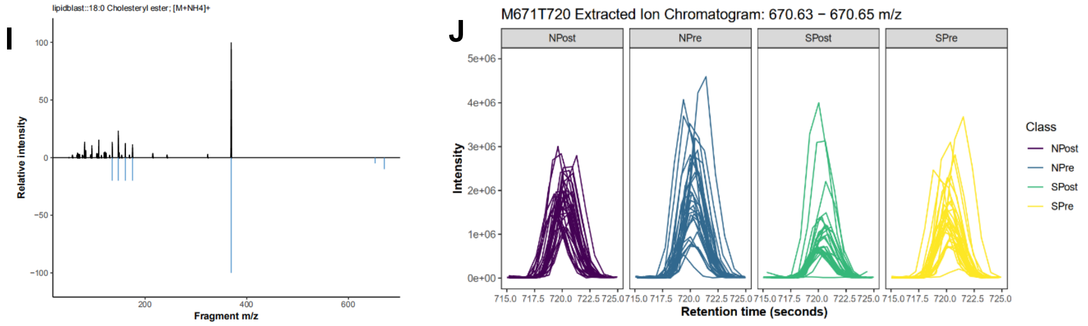

Figure S3. MS/MS annotated spectra and extracted ion chromatogram of 5 final biomarkers from machine learning workflow.
